# *Aggregatibacter actinomycetemcomitans* Outer Membrane Proteins 29 and 29 Paralogue Induce Evasion of Immune Response

**DOI:** 10.3389/froh.2022.835902

**Published:** 2022-02-03

**Authors:** Maike Paulino da Silva, Viviam de Oliveira Silva, Silvana Pasetto, Ellen Sayuri Ando-Suguimoto, Dione Kawamoto, Gardênia Márcia Silva Campos Mata, Ramiro Mendonça Murata, Marcia Pinto Alves Mayer, Casey Chen

**Affiliations:** ^1^Departamento de Microbiologia, Instituto de Ciências Biomédicas, Universidade de São Paulo, São Paulo, Brazil; ^2^Division of Periodontology, Diagnostic Sciences and Dental Hygiene, Ostrow School of Dentistry of University of Southern California, Los Angeles, CA, United States; ^3^Centro Universitário Atenas- UniAtenas, Paracatu, Brazil; ^4^Department of Comprehensive Oral Health, Adams School of Dentistry, University of North Carolina, Chapel Hill, NC, United States; ^5^Instituto de Alimentação e Nutrição, Centro Multidisciplinar UFRJ-Macaé, Universidade Federal do Rio de Janeiro, Macaé, Brazil; ^6^Department of Foundational Sciences, School of Dental Medicine of University of East Carolina University, Greenville, NC, United States

**Keywords:** *Aggregatibacter actinomycetemcomitans*, outer membrane protein 29, outer membrane protein 29 paralogue, aggressive periodontitis, gingival epithelial cells, inflammatory response, virulence factors

## Abstract

*Aggregatibacter actinomycetemcomitans* (*Aa*) is abundant within the microbial dysbiotic community of some patients with periodontitis. *Aa* outer membrane protein 29 (OMP29), a member of the OMPA family, mediates the invasion of *Aa* to gingival epithelial cells (GECs). This study evaluated the effect of OMP29 and its paralogue OMP29^par^ on the response of GECs to *Aa*. The *omp29* or/and *omp29*^*par*^ deletion mutants AaΔ29, AaΔ29P, and AaΔ29Δ29P were constructed, and recombinant *Aa* OMP29^His^ was obtained. Microarray analysis and the evaluation of *cxcl-8* gene expression were performed to examine the response of GECs line OBA-09 to *Aa* and its mutants. The expression of *cxcl-8* and its product CXCL-8 was examined in LPS-stimulated OBA-09 cells with *Aa* OMP29^His^. Proteomics analysis showed that the deletion of *omp29* led to overexpression of both OMP29^par^ and another membrane protein OMP39, the expression of which was further increased in AaΔ29Δ29P. OBA-09 cells challenged with AaΔ29Δ29P exhibited a higher expression of *cxcl-8* in comparison to wildtype *Aa* strain AaD7S or single-deletion mutants AaΔ29 or AaΔ29P. LPS-stimulated OBA-09 cells challenged with *Aa* OMP29^His^ showed reduced expressions of *cxcl-8* and its product CXCL-8. OBA-09 cells challenged with AaΔ29Δ29P in comparison to *Aa* strain AaD7S resulted in higher expressions of genes involved in apoptosis and inflammatory response such as *bcl2, birc3, casp3, c3, ep300, fas, fosb, grb2, il-1*α*, il-1*β*, il-6, cxcl-8, nr3c1, prkcq, socs3*, and *tnfrsf1*β and reduced expressions of *cd74, crp, faslg, tlr1*, and *vcam1*. The results suggested a novel strategy of *Aa*, mediated by OMP29 and OMP29^par^, to evade host immune response by inhibiting CXCL-8 expression and modulating the genes involved in apoptosis and inflammatory response in GECs. Pending further confirmation, the strategy might interfere with the recruitment of neutrophils and dampen the host inflammatory response, leading to a more permissive subgingival niche for bacterial growth.

## Introduction

*Aggregatibacter actinomycetemcomitans (Aa*) is associated with a rapidly progressing form of periodontitis in young subjects, previously known as localized aggressive periodontitis [1–7]. Recent microbiome data confirmed this association and indicated that *Aa* is 50 times more abundant in periodontal pockets of Grade C/molar–incisor pattern periodontitis (GC/MIP) patients than in subgingival sites of healthy subjects [8]. *Aa* can invade non-phagocytic cells, such as gingival epithelial cells, escape from the vacuole, multiply, exit, and spread to adjacent cells [9]. *Aa* interacts with epithelial cells through adhesins such as OMP100 [10]. In contrast, invasion is mediated by the outer membrane protein 29 (OMP29) *via* the focal adhesion kinase (FAK) pathway [11]. Furthermore, OMP29 causes apoptosis of gingival epithelial cells (GECs) [12]. The OMP29 and its paralogue can inhibit the classical and mannose-binding lectin complement activation by binding to C4-binding protein [13].

The OMP29 belongs to the OMPA-like family and it is one of the six major OMPs recognized by antibodies in the serum of patients with periodontitis [14]. Furthermore, immunoglobulin G (IgG) titers against OMP29 and *Aa* serotypes b and c are reduced after successful periodontal treatment in aggressive periodontitis patients [15].

The GECs are the first barrier faced by the subgingival microbial community and play a key role in defense by detecting pathogen-associated molecular patterns (PAMPs) and triggering the immune response to eliminate the infecting pathogen [16]. However, pathogenic bacteria manipulate the host response to promote survival by activating or inhibiting different signaling pathways. Several members of the OMPA-like family were shown to contribute to immune evasion of virulent bacteria through the down-regulation of expression of inflammatory mediators such as CXCL-8 [17, 18]. Other proteins of the OMPA family, such as OMPA from *Acinetobacter* spp. stimulates the *cxcl-8* expression [19] and *Aa* OMP29 seems to increase CXCL-8 production by human GECs [20]. However, little is known about the role of *Aa* OMP29 in host response.

Therefore, we aimed to evaluate the role of OMP29 on the immune response of GECs after interaction with *Aa*. The OMP29 deletion mutants and recombinant OMP29 interacted with GECs and the response was evaluated at gene and protein differential expression levels.

## Materials and Methods

### Strains, Plasmids, Media, and Growth Conditions

The bacteria strains, plasmids, and primers used in this study are described in Table 1. The *Aa* D7S-1 wildtype strain [21, 22], designated AaD7S, was used to construct the mutants in *omp29* and *omp29* paralogue (*omp29*^*par*^) genes. The *Aa* HK1651 wildtype strain (Genbank Access AY 262734) was used to obtain the OMP29 recombinant protein. *Aa* strains were grown in a humidified 5% CO_2_ atmosphere at 37°C (Shel Lab, OR, United States), in modified Trypticase Soy Broth (mTSB) containing 3% trypticase soy broth and 0.6% yeast extract, or on mTSB agar [mTSB with 1.5% agar (Oxoid Ltd, Basingstoke, Hampshire, England)].

**Table 1:** Strains, plasmids, and primers used in this study.

**Strain**	**Description**	**Source**
D7S-1 (AaD7S)	*Aa* serotype a	[21, 22]
AaΔ29	*Aa* D7S-1, Δ*omp29*	This study
AaΔ29P	*Aa* D7S-1, Δ*omp29^*par*^*	This study
AaΔ29Δ29P	*Aa* D7S-1, Δ*omp29* Δ*omp29^*par*^*	This study
H5P-1	*Aa* serotype a	[23]
HK1651	*Aa* seroype b, highly leukotoxic JP2-like clone	[23]
ANH9381	*Aa* serotype b	[23]
D11S-1	*Aa* serotype c	[23]
D17P-2	*Aa* serotype c	[23]
*E. coli* BL21(DE3)	F^−^*omp*T *hsd*S_B_ (${\text{r}}_{\text{B}}^{-}$, ${\text{m}}_{\text{B}}^{-}$) *ga*l *dcm* (DE3)	ThermoFisher, Carlsbad, CA, United States
*E. coli* DH5-α	F^−^ϕ80*lac*ZΔM15 Δ(*lac*ZYA-*arg*F)U169 *rec*A1 *end*A1 *hsd*R17(rK–, mK+) *pho*A *sup*E44 λ- *thi*-1 *gyr*A96 *rel*A1	ThermoFisher, Carlsbad, CA, United States
**Plasmid**	**Description**	**Source**
plox2-Spe^r^	pBluescript II KS derivative containing a *spe* cassette flanked by *loxP* sites (ATGgATGCa), Amp^r^, Spec^r^	[24]
pAT/Cre	pPK1 derivative containing the *cre* gene and *tet(O)* genes, Tc^r^	[25]
pCR^®^4-TOPO^®^	cloning vector, Amp^r^, Kn^r^	Invitrogen, Carlsbad, CA, United States
pTOPOomp29SPS	pCR4-TOPO with *omp29* without signal peptide in *BamHI* and *XhoI* sites	This study
pET28B	T7 expression vector, C-terminal 6x histidine tag, Kn^r^	Merck, Darmstadt, Germany
pET28B/*omp29*	pET28B with *omp29* without signal peptide in *BamHI* and *XhoI*	This study
**Primer**	**Sequence 5** **′** **−3** **′**	**Reference**
Omp29ParalUp-F (537 bp)	ACAAGCAAAATATAATGAAGCACAGG	This study
Omp29ParalUp-R (537 bp) *DraIII*	TTCACGTGGTGTCTCCTATATTATTAATTTG	This study
Omp29ParalDw-F (503 bp) *DraIII*	TACACGTGGTGTTTAATTGAAGAATAAATAAG	This study
Omp29ParalDw-R (503 bp)	CACCACAAAAGTAGTCTTACAATCC	This study
Omp29Up-F (528 bp)	AAGTGTTGTCGGTACAGAGCATTC	This study
Omp29Up-R (528 bp) *DraIII*	TTCACGTGGTGGATCCTCTATTAATTAGTC	This study
Omp29Dw-F (610 bp) *DraIII*	AACACGTGGTGTGTTAATTGTTAGCAAATAG	This study
Omp29Dw-R (610 pb)	GTTTTAAGCTCACCTTGTTGGTACATTTC	This study
T7 Universal	TAATACGACTCACTATAGGG	Merck, Darmstadt, Germany
T7 Terminator	GCTAGTTATTGCTCAGCGG	Merck, Darmstadt, Germany
pETOMP29-F (1051 bp) *BamHI*	TGAAAAGGGATCCAATCGCATT	This study
pETOMP29-R (1051 bp) *XhoI*	AACTCGAGAATTATTTACTACCG	This study
CXCL-8 (147 bp)	QuantiTect Primer Assay Dr_il8_1_SG	(Qiagen Cat # QT02108190; Valencia, CA, United States).

*The underlined sequences correspond to restriction enzymes cleavage sites*.

The plasmids used in this study were replicated in *Escherichia coli* strains DH5-α and *E. coli* BL21(DE3) (Invitrogen, Carlsbad, CA, United States) were cultured by standard methods [26].

Spectinomycin (50 μg/ml, Sigma–Aldrich, St. Louis, MO, United States), tetracycline (4 μg/ml, Sigma–Aldrich), ampicillin (100 μg/ml, Sigma-Aldrich), kanamycin, (50/100 μg/ml, Sigma–Aldrich), and chloramphenicol (50 μg/ml, Sigma–Aldrich) were added to the media when selection of mutants or transformants were needed.

The growth curve was determined in mTSB with the addition of mineral oil to create a microaerophilic condition in 100-well HoneyComb plates (Bioscreen Automation for Microbiology, NJ, United States). The optical density (OD) at 420-580 nm was monitored in a spectrophotometer (Growth Reader Bioscreen C, Oy Growth CurvesAb Ltd., Bioscreen C Type FP-1100-C, FIN-21280 Raisio, Finland) in 1 h intervals for 16 h at 37°C, under agitation. The growth rate was calculated according to the formula: $\frac{\mu \;=\;Ln\frac{N}{N0}}{t}$, where *N* and *N*_0_, respectively, correspond to the initial and final *OD*_420−580_ at the exponential growth phase and *t* is the time-course of the growth curve.

### DNA Manipulation

Genomic DNA of *Aa* D7S-1 and *Aa* HK1651 was obtained by using QIAamp DNA mini kit (Qiagen, Valencia, CA, United States). Plasmids DNA was obtained by using QIAprep Spin Miniprep kit (Qiagen). *E. coli* DH5-α and BL21(DE3) strains (Invitrogen, Carlsbad, CA, United States) were transformed with pCR 4-TOPO (Invitrogen) and vector pET28B vectors (pET Expression System 28, Novagen), respectively, by electroporation (MicroPulser, BioRad, Hercules, CA, United States). Restriction enzymes, T4 ligase, and Taq DNA polymerase were purchased from New England BioLabs Inc. (Ipswich, MA, United States). PCR was performed with Taq high-fidelity polymerase (Invitrogen), and its products were purified using QIAquick PCR purification and QIAquick gel extraction kits (Qiagen).

### Obtaining *Aa Omp29* and *Omp29^*par*^* Defective Strains

Deletion mutants in *omp29, omp29*^*par*^, and both genes were obtained by using the loxP/Cre system [21, 26]. Briefly, the spectinomycin cassette loxP-Specr-loxP was obtained by restriction digestion of plox2/Specr vector [24] with *DraIII*. The *omp29* or *omp29*^*par*^ flanking regions were obtained by amplification with primer Omp29Up-F, Omp29Up-R, Omp29Dw-F, Omp29Dw-R, and Omp29ParalUp-F, Omp29ParalUp-R, Omp29ParalDw-F, and Omp29ParalDw-R, respectively, and digested with *DraIII*. This product was then ligated to the loxP-Specr-loxP fragment and transformed into the competent AaD7S strain.

Transformants were selected in TSYE agar with spectinomycin. The allelic replacement of the target gene was confirmed by PCR and sequencing. The removal of loxP-Specr-loxP of strains was done by transformation with pAT/Cre vector [25], which encodes Cre recombinase.

The *omp29* and *omp29*^*par*^ deficient mutants of AaD7S were named AaΔ29 and AaΔ29P. The double deletion mutant (referred to as AaΔ29Δ29P) was constructed by the deletion of *omp29*^*par*^ from the AaΔ29 strain following the procedure described above, with the exception that the spectinomycin resistance cassette in *omp29*^*par*^ was not removed.

The success of the mutation strategy was confirmed by PCR and sequencing (data not shown) using the following primers: Omp29Up-R, Omp29Dw-F, Omp29ParalUp-R, and Omp29ParalDw-F, which annealed downstream and upstream of *omp29* and *omp29*^*par*^, respectively.

### *Aa* Recombinant OMP29

The Signal 3.0 Server program was used to predict the sequence that encodes the signal peptide of *omp29*. Based on this prediction, the pET OMP29 F and R primers with *BamHI* and *XhoI* restriction sites were used to amplify *omp29* from *Aa* HK1651 (Genbank accession number AY 262734), constructed previously in pCR4-TOPO vector (designated as pTOPOomp29SPS). After digestion, the *omp29* amplicon (without the signal peptide) was ligated to pET28B vector (pET Expression System 28, Novagen) and the resulting pET28B/*omp29* vector was transformed into *E. coli* BL21 (DE3). The recombinant plasmids were confirmed by T7 Universal and Terminator primer sets for sequencing.

### Expression and Purification of *Aa* OMP29^His^

The OMP29 was expressed by pET28B/*omp29 E. coli* BL21 (DE3) following the manufacturer's instructions (New England Biolabs, Ipswich, MA, United States). A single colony of pET28B/*omp29 E. coli* BL21 (DE3) was inoculated into LB broth with kanamycin (50 μg/ml) (Sigma–Aldrich, St. Louis, MO, United States) and incubated under agitation at 120 rpm for 16 h at 37°C. Cells were transferred to fresh LB broths, and incubated under agitation at 250 rpm at 37°C until O.D. 600 nm ≈ 1 was reached. Expression of *Aa* OMP29^His^ was induced by adding 1 mM of isopropyl β-D-thiogalactoside (IPTG, Sigma–Aldrich, St. Louis, MO, United States) at 37°C and the recombinant protein was observed after 2 h in culture precipitate. Cells were then lysed [27] and *Aa* OMP29^His^ in cells lysate supernatant was purified by affinity chromatography using nickel chelating resin (Ni-NDA, Invitrogen).

### Serum Anti-*Aa* OMP29^His^

Balb/c mice were immunized subcutaneously with 50 μg of purified *Aa* OMP29^His^ with adjuvant aluminum hydroxide (1:1) in a volume of 200 μl. Booster injections were performed after 7 days, 3, 4, 6, and 7 weeks of the initial challenge. The titers of anti-*Aa* OMP29^His^ antibodies were determined by immunoenzymatic assay (ELISA). Statistical analysis was performed in GraphPad Prism version 6.0 (GraphPad Software). This assay was approved by the Ethics and Research Committee on Animals of the Institute of Biomedical Sciences of the University of São Paulo under the protocol number 007/11.

### Sodium Dodecyl Sulfate–Polyacrylamide Gel Electrophoresis

The bacterial outer membrane extracts (OMEs) of AaD7S and its mutants AaΔ29, AaΔ29P, and AaΔ29Δ29P were obtained as previously described [28], and outer membrane proteins (OMPs) were resolved by standard SDS-PAGE (10% acrylamide) [29]. Purified *Aa* OMP29^His^ was also detected after SDS-PAGE using the Bolt Bis-Tris Plus gel (Life Technologies, Carlsbad, CA, United States) and the Bolt Mini Gel Tank (Life Technologies). The gels were stained with Colloidal blue (Life Technologies). OMP29 protein has the approximate size of 29 kDa, however, appears as a 34 kDa band when denatured [14].

### Proteomic Analysis

After SDS-PAGE, the OMPs corresponding bands of AaD7S and its mutants AaΔ29, AaΔ29P, and AaΔ29Δ29P were excised, eluted with 1% acetic acid/H_2_O, and analyzed in the mass spectrometer (LTQ Orbitrap Hybrid Mass Spectrometers), and raw data were processed using Proteome Discoverer software (Thermo Scientific). Twelve samples were analyzed. Individual proteins were identified and ranked by protein scores (calculated using SEQUEST algorithm on Proteome Discoverer), which is the sum of all peptide Xcorr values above the specified score threshold. The score threshold is calculated as follows: 0.8 + peptide charge × peptide relevance factor where peptide relevance factor is a parameter with a default value of 0.4. The peptide spectrum matches were used as a surrogate for the relative amounts of individual proteins in each sample.

### Immunoblotting

Following electrophoresis, *Aa* OMP29^His^ was transferred to a nitrocellulose membrane using an iBlot 2 Dry Blotting System kit (Life Technologies). The membrane was blocked with 5% skimmed milk in TBS-T (20 mM Tris-HCl pH 7.6, 0.8% NaCl and 0.1% Tween 20). Anti-OMP29^His^ polyclonal serum obtained in Balb/c mice and serum of aggressive periodontitis patients colonized by *Aa* [15] were used as primary antibodies at 1:2,000 dilution in separate membranes. Secondary antibodies consisted of goat anti-mice IgG peroxidase conjugated (Amershan Bioscience, Amershan, Little Chalfont, United Kingdom) or goat anti-human IgG peroxidase conjugated (ThermoFisher, Carlsbad, CA, United States) diluted to 1:10,000. *Aa* OMP29^His^ bands were detected by using the SuperSignal West Pico Chemiluminescent Substrate (ThermoFisher, Carlsbad, CA, United States).

### Interaction Assays Between GEC and *Aa* Strains

OBA-09 cells [30], immortalized GEC lineage, were grown at 37°C in humidified 5% CO_2_ atmosphere in collagen type I pre-coated bottles (BD Bioscience, San Jose, CA, United States) containing complete Keratinocyte Serum-Free Medium (KSFM) (ThermoFisher, Carlsbad, CA, United States) supplemented with insulin, epidermal growth factor, and fibroblast growth factor (complete KSFM) according to the manufacturer's recommendation (ThermoFisher, Carlsbad, CA, United States). After confluent growth, OBA-09 cells were scraped, centrifuged at 593 × *g* for 10 min at 4°C, resuspended in complete KSFM, added to wells of plates at 1 × 10^5^ cell/ml, and incubated for 16 h.

The interaction assay was performed as previously described [11]. Log phase cultures of AaD7S and its mutants AaΔ29, AaΔ29P, and AaΔ29Δ29P were adjusted to 1 × 10^8^ cell/ml in a complete KSFM and added to OBA-09 cells at a multiplicity of infection of 1:1,000 (OBA-09:bacteria).

After 4 h of interaction, total RNA was isolated using the RNeasy Mini Kit (Qiagen; Valencia, CA, United States) and converted into cDNA using QuantiTect Reverse Transcription Kit (Qiagen).

Quantitative Real-Time Reverse Transcription-Polymerase Chain Reaction (qRT-PCR) analysis was carried out to evaluate *cxcl-8* relative expression. The reaction was performed using the StepOnePlus Real-Time PCR System (Thermo Fisher Scientific, Rockford, United States) using the QuantiTect SYBR Green PCR and QuantiTect Primer Assay Cat # QT02108190 (Qiagen; Valencia, CA, United States).

The gene expression of inflammatory response was analyzed by Microarray using the Prime PCR Pathway Plate/Acute Inflammation Response H96 system (Bio-Rad; Hercules, CA, United States), according to the manufacturer's instructions and performed in CFX96 Touch Real-Time PCR Detection Systems (Bio-Rad).

Both Microarray and qRT-PCR data resulted in CT values for each sample. Gene dosages were obtained using the 2^−Δ*ΔCT*^ Livak method [31, 32], considering glyceraldehyde-3-phosphate dehydrogenase (*gapdh*) as a reference gene and co-culture OBA-09 cell with AaD7S strain as the control. Statistical analysis was performed in GraphPad Prism version 6.0 (GraphPad Software).

### Interaction Assays Between GEC and *Aa* OMP29^His^

The confluent monolayers of OBA-09 cell, as previously described, were added to 24 well/plates (ThermoFisher, Carlsbad, CA, United States) and challenged with *E. coli* serotype O111:B4 LPS (500 ng/ml; Sigma–Aldrich). Then, aliquots of 1 μg/ml and 10 μg/ml of *Aa* OMP29^His^ were added to each well. After 4 h of interaction, levels of CXCL-8 in free cell supernatant were established by Bio-Plex Pro Human Chemokine assay kit (Bio-Rad, United States), following the manufacturer's instructions. Those levels were determined by comparison to the standard curve (mean of fluorescence intensity vs. pg/ml), using Bio-Plex software manager 4.0. The results were shown in percentage with CXCL-8 concentration (pg/ml) of the 4 h cultured with OBA-09 being 100% and statistical analysis was performed in GraphPad Prism version 6.0 (GraphPad Software).

### Sequence Analysis

Multiple sequence alignment was performed with Clustal Omega (https://www.ebi.ac.uk/Tools/msa/clustalo/) using the default setting. The amino acid sequences of *Aa* OMP29 and OMP29^par^ of *Aa* strains HK1651, ANH9381, D7S-1, H5P-1, D11S-1, and D17P-2 (Table 1) were identified and downloaded from our annotated database at http://expression.washington.edu/genetable/script/gene_table_viewer [23].

## Results

### OMP29 and OMP29^par^ Protein Sequence

The sequence alignments of OMP29 and OMP29^par^ of representative strains of serotype a, b, and c are provided in Supplementary Figure 1. The OMP29 is 346 aa long, and the OMP29^par^ is 356 aa long. Each orthologous protein demonstrates 99% amino acid sequence identity among strains. The OMP29 and OMP29par also share approximately 75% amino acid sequence identify.

### Human and Murine Sera Recognize OMP29 Recombinant

The recombinant protein *Aa* OMP29^His^ was obtained, purified, and confirmed by its reaction with mice anti-*Aa* Omp29^His^ IgG polyclonal and by human aggressive periodontitis sera in a Western-blot assay (Supplementary Figure 2).

### Alteration on OMP Profile of *Aa* After Knocking out *Omp29* and/or *Omp29^*par*^*

**Figure 1:**
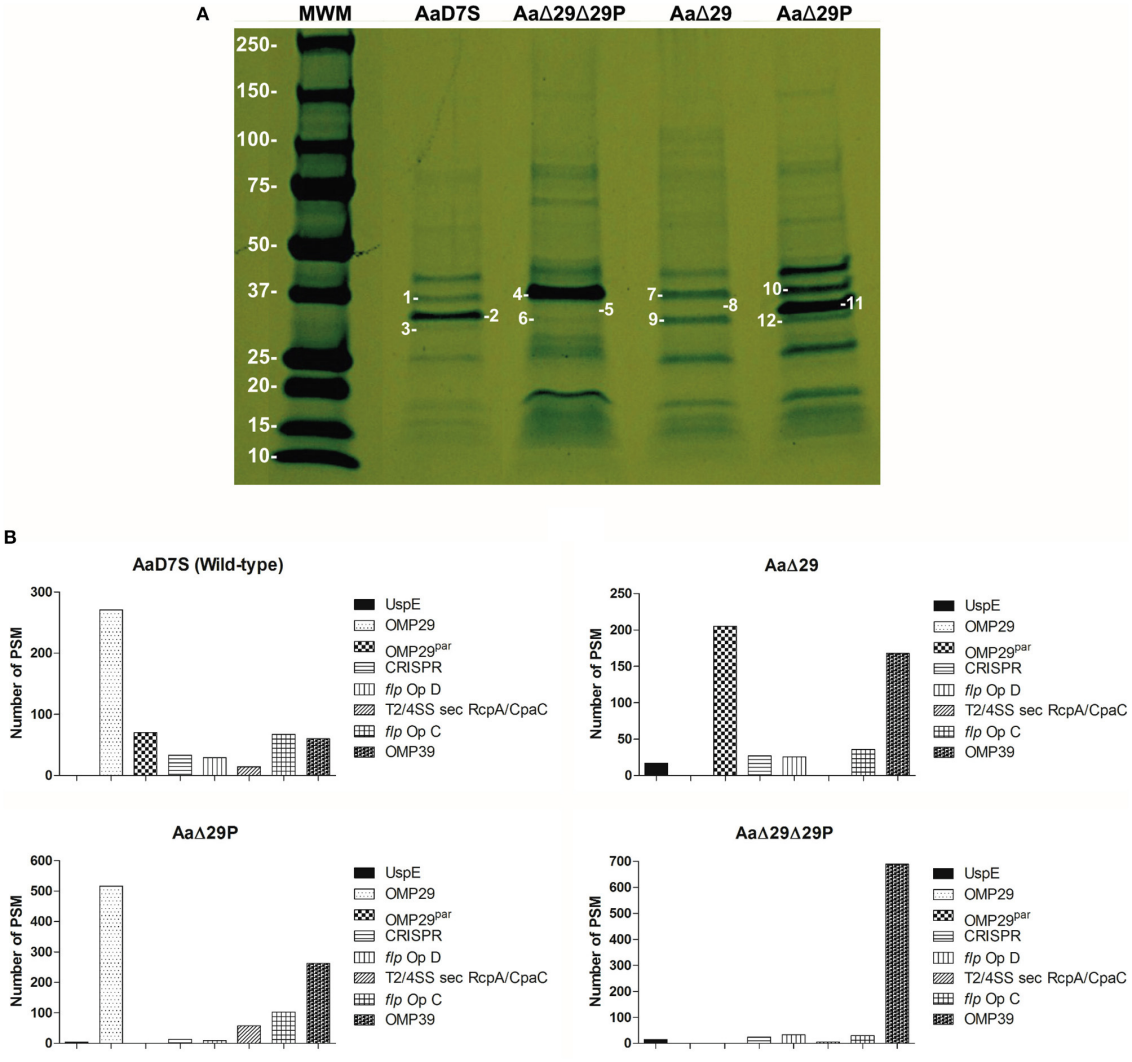
Outer membrane extracts profile of *Aa* D7S-1 strains. **(A)** Outer membrane protein profiles of *Aa* grown until mid-log phase were determined by SDS-PAGE analysis: MWM: Molecular Weight Marker—Precision Plus Protein Standard, Bio-Rad, in kDa. Gel slices numbered 1-12 showed the following OMPs: AaD7S, 1-OMP39, 2-OMP29; 3-OMP29^par^; AaΔ29Δ29P, 4-OMP39, 5-OMP29 absent; 6-OMP29^par^ absent; AaΔ29, 7-OMP39, 8-OMP29 absent; 9-OMP29^par^; AaΔ29P, 10-OMP39, 11-OMP29; 12-OMP29^par^ absent). Photograph of a representative gel of at least two independent assays. **(B)** Proteomic analysis of detected peptides or peptide spectrum matches (PSM) for the 8 major proteins: (Universal stress protein UspE, OMP29, OMP29^par^, CRISP-associated protein (CRISP), *flp* operon protein D (*flp* Op D), type II/IV secretion system secretin RcpA/CpaC (T2/4SS sec RcpA/CpaC), and *flp* operon protein (flp Op C) and OMP39) after combining gel #1-3, gel #4-6, gel #7-9, and gel #10-12, to determine whether some proteins were differentially expressed in AaD7S and its mutants AaΔ29Δ29P, AaΔ29, and AaΔ29P, respectively. AaD7S, *Aa* D7S-1 wildtype strain; AaΔ29, *Aa* D7S-1 *omp29* mutant strain; AaΔ29P, *Aa* D7S-1 *omp29*^*par*^ mutant strain; AaΔ29Δ29P, *Aa* D7S-1 *omp29* and *omp29*^*par*^ mutant strain.

*Aa* mutants in *omp29, omp29*^*par*^ and in both genes were constructed and characterized. All strains presented similar growth rates in mTSB medium (Supplementary Figure 3). Analysis of OME revealed differences in the protein profile between the wildtype and the *omp29* and *omp*29^*par*^ deletion mutants (Figure 1). To better assess the profile, the distinct bands were excised from the gel and evaluated by mass spectrophotometry. The identity and the relative amounts of individual proteins differentially expressed were determined (Supplementary Table 1).

Proteomic analysis indicated that OMP29 and OMP29^par^ were simultaneously expressed by the wildtype strain AaD7S, whereas the target proteins were absent in AaΔ29, AaΔ29P, and AaΔ29Δ29P. The deletion of *omp29* led to overexpression of at least 3 times of both OMP29^par^ and OMP39. The deletion of OMP29^par^ resulted in a 2 times increase in OMP29 levels and 4 times increase in OMP39 levels (Figure 1). Furthermore, the double deletion in strain AaΔ29Δ29P resulted in the overexpression of OMP39 (11 times increase compared to the AaD7S). The relative expression levels of other proteins also changed in the mutants, although not as pronounced as seen for OMP39. The physiological significance of these changes is unknown but may represent a compensatory mechanism.

### *Aa* OMP29 Represses CXCL-8

**Figure 2:**
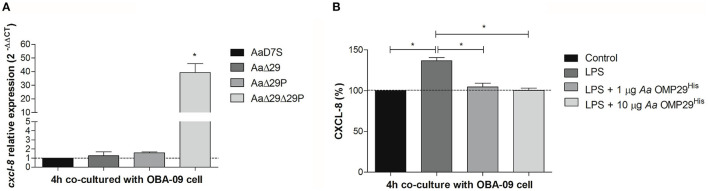
Chemokine CXCL-8 expression. **(A)** *cxcl-8* relative transcription. *Aa* wildtype strain (AaD7S) and its mutant strains (AaΔ29, *Aa* D7S-1 *omp29* mutant strain; AaΔ29P, *Aa* D7S-1 *omp29*^*par*^ mutant strain; AaΔ29Δ29P, *Aa* D7S-1 *omp29* and *omp29*^*par*^ mutant strain) were grown in mTSB until mid-exponential phase and then co-cultured with confluent monolayers of OBA-09 cells for 4 h in complete KSF medium. After the RNA extraction and cDNA synthesis, qRT-PCR was performed and relative mRNA levels of *cxcl-8* were evaluated using *gapdh* as reference. OBA-09 cell was co-cultured with AaD7S as a control (dotted line). *One-way ANOVA followed by Dunnett's post-test (significant, *p* < 0.05). **(B)** CXCL-8 production. OBA-09 cells were grown in KSF medium and challenged with *E. coli* serotype O111:B4 LPS followed by treatment with 1 μg/ml and 10 μg/ml *Aa* OMP29^His^ for 4 h. CXCL-8 levels in cell supernatants were evaluated by ELISA, and data were reported as the percentages of CXCL levels in relation to non-inoculated cells (control) (dotted line). LPS, lipopolysaccharide; *Aa* OMP29^His^, OMP29 recombinant protein of *Aa* HK1651. *One-way ANOVA followed by Tukey's post-test (significant, *p* < 0.05).

CXCL-8 plays a significant role in the pathogenesis of periodontitis. In this study, we aimed to confirm the effect of OMP29 and OMP29^par^ on the inhibition of *cxcl-8* transcription. The effect of the 4 h interaction of *Aa* wildtype and its mutants with GECs on *cxcl-8* transcription was evaluated by RT-qPCR. The AaΔ29Δ29P mutant promoted a significant increase in *cxcl-8* transcripts levels in GECs compared to the wildtype. However, the deletion of *omp29* or *omp29*^*par*^ did not result in *cxcl-8* regulation, suggesting functional redundancy of OMP29 and OMP29^par^ in the regulation of *cxcl-8* expression (Figure 2A).

To determine whether the effect on *cxcl-8* expression was due to the compensatory expression of other proteins such as OMP39 in the mutants, we investigated the interaction between OBA-09 cell and *Aa* OMP29^His^ (Figure 2B). *E. coli* LPS was used to trigger CXCL-8 expression in OBA-09 cells [33, 34], and cells were challenged with 1 or 10 μg/ml of *Aa* OMP29^His^. After 4 h of interaction, the expression of CXCL-8 was determined at the protein level. The analyses indicated that both concentrations of *Aa* OMP29^His^ could inhibit the transcription of *cxcl-8*, resulting in reduced CXCL-8 levels in OBA-9 cells supernatants compared to control cells (Figure 2B).

### *Aa* OMP29 and OMP29^par^ Alter the Response of GECs

After observing the effect of OMP29 on CXCL-8 expression, we explored the gene expression profile of host acute inflammatory response to AaD7S and its mutants. The results showed modulatory effects (at least two-fold) of 21 genes (Table 2; for gene function, see Supplementary Table 2). Sixteen genes had increased (*bcl2, birc3, casp3, c3, ep300, fas, fosb, grb2, il-1*α*, il-1*β*, il-6, cxcl-8, nr3c1, prkcq, socs3*, and *tnfrsf1*β) and five had genes decreased (*cd74, crp, faslg, tlr1*, and *vcam1*) expression in OBA-09 cells co-cultured with AaΔ29Δ29P strain compared to AaD7S. The data suggested a potential role of OMP29 and its paralogue in the host response of GECs. Nevertheless, these findings suggested a possible influence of OMP29 and its paralogue in anti-apoptotic (e.g., *bcl2, birc3, grb2, prkcq*) and pro-apoptotic (e.g., *casp3, c3, ep300, fosb, nr3c1, crp*, and *socs3*) pathways. Furthermore, the interaction with the strain AaΔ29Δ29P down-regulated transcription of *cd74, faslg, tlr1*, and *vcam1* and up-regulated *fas* and *tnfrsf1*β. The *faslg* and *fas* also participate in pro-apoptotic pathways. Finally, interaction with the strain AaΔ29Δ29P upregulated transcription of *il-1*α, *il-1*β, *il-6, cxcl-8*, and *tnfrsf1*β, encoding cytokines and chemokines involved in the inflammatory response. Notably, the effect promoted by the absence of OMP29 or OMP29^par^ was not as intense as observed for the double mutant, possibly due to the compensation mechanism induced by their absence (Table 2). Alternatively, the effects described above may be due to yet to be characterized pleiotropic effects of the deletion of *omp29* and *omp29*^*par*^.

**Table 2:** Inflammatory response of OBA-09 cell after 4 h of interaction with *Aa* strains.

**Gene**	**Mean of relative expression (2**^**−***ΔΔCT*****^ method) in each interaction assay^*^			
	**OBA-09 + AaD7S (control)**	**OBA-09 + AaΔ29**	**OBA-09 + AaΔ29P**	**OBA-09 + AaΔ29Δ29P**
*bcl2*	1.00	42.91^**^	84.45^**^	54.82^**^
*birc3*	1.00	5.04^**^	10.13^**^	44.02^**^
*casp3*	1.00	27.16^**^	27.67^**^	49.18^**^
*c3*	1.00	1.84	1.01	14.62^**^
*ep300*	1.00	48.95^**^	23.92^**^	33.59^**^
*fas*	1.00	1.39	1.46	7.16^**^
*fosb*	1.00	186.11^**^	179.77^**^	242.20^**^
*grb2*	1.00	5.25^**^	3.68^**^	4.58^**^
*il-1α*	1.00	0.22^**^	0.97	6.96^**^
*il-1β*	1.00	0.33^**^	0.67	14.42^**^
*il-6*	1.00	1.86	1.36	50.56^**^
*cxcl-8*	1.00	0.35^**^	0.82	11.74^**^
*nr3c1*	1.00	3.61^**^	3.81^**^	4.76^**^
*prkcq*	1.00	2.42^**^	2.17^**^	13.27^**^
*socs3*	1.00	1.56	1.87	10.41^**^
*tnfrs1β*	1.00	1.08	0.91	11.88^**^
*cd74*	1.00	0.43^**^	0.47^**^	0.09^**^
*crp*	1.00	0.45^**^	0.62	0.00^**^
*faslg*	1.00	0.46^**^	0.59	0.06^**^
*trl1*	1.00	0.39^**^	0.57	0.06^**^
*vcam1*	1.00	0.69	0.89	0.16^**^

* *Microarray analysis using Prime PCR Pathway Plate/Acute Inflammation Response H96 system (Bio-Rad; Hercules, CA, United States). The gapdh was used as a reference gene and OBA-09 cell was co-cultured with AaD7S as a control. The experiment was done once with triplicate. AaD7S: Aa D7S-1 wildtype strain; AaΔ29, Aa D7S-1 omp29 mutant strain; AaΔ29P, Aa D7S-1 omp29^par^ mutant strain; AaΔ29Δ29P, Aa D7S-1 omp29 and omp29^par^ mutant strain*.

** *At least two increasing or decreasing fold changes in relative transcription in GECs were challenged with the wildtype strain*.

## Discussion

To improve our understanding of the mechanisms involved in periodontitis associated with *Aa*, we aimed to evaluate the effect of *Aa* OMP29 on the interaction with gingival epithelial cells. Outer membrane protein 29 and the other five outer membrane proteins of *Aa* (OMP100, OMP64, OMP39, OMP16, and OMP18) were recognized by antibodies in the serum of patients with periodontitis of high rate of progression in young subjects [9]. OMP29 is an OMPA-like protein and its paralogous OMP29^par^ was identified in our study and a in recently published article [13].

We have noticed increased and compensatory expressions of other OMPs associated with the deletion of *omp29* and *omp29*^*par*^. For example, the deletion of *omp29* led to an over-expression of OMP29^par^, and the deletion of *omp29* or *omp29*^*par*^ led to an over-expression of OMP39. We have also noted the changes in the transcription profiles of GECs challenged with the defective mutants.

The more robust alterations in mRNA levels were observed for the double mutant than when GECs were challenged with the single mutant. This is likely due to the functional redundancy and compensatory expression of OMP29 or OMP29^par^ in the corresponding single-deletion mutants, which may exert similar effects to GECs as the wildtype AaD7S.

The study examined the role of OMP29 in the expression of *cxcl-8* in GECs. We focused on CXCL-8 since this chemokine is associated with inflammatory tissue destruction in periodontitis and periodontal tissue homeostasis. The expression of *cxcl-8* was higher in OBA-09 cells challenged with AaΔ29Δ29P than with the wildtype AaD7S. To rule out the effects associated with the compensatory expressions of other proteins such as OMP39, we tested the recombinant *Aa* OMP29^His^ and demonstrated its ability to suppress *cxcl-8* transcription and CXCL-8 production in LPS-stimulated OBA-9 cells. The results suggested that OMP29 and OMP29^par^ were involved in the inhibition of *cxcl-8*. Our results were in disagreement with another study showing the induction of CXCL-8 by OMP29 in gingival epithelial cells [20]. Previous studies extracted OMP29 directly from *Aa* outer membrane, which may contain contaminants that affect the outcomes of the experiments [11–14, 20, 35].

Previous data reported that *Aa* OMP29 promotes apoptosis in GECs *via* TGF-βR/smad2 pathway by binding fibronectin (Fn) to ease Fn/integrinβ1/FAK signaling-dependent TGF-β release from the extracellular matrix [12]. The primary mechanism of defense of the host cell against intracellular bacteria is the induction of apoptosis. However, obligate intracellular pathogens such as *Chlamydia* [36] may have anti-apoptotic mechanisms to promote their survival in the intracellular environment. This study showed that the expression of several genes involved in apoptotic pathways was altered in OBA-09 cells when co-cultured with the double mutant strain AaΔ29Δ29P, suggesting that OMP29 or OMP29^par^ may regulate apoptosis of GECs, influencing the intracellular survival of *Aa*. However, both pro-apoptotic genes (*casp3, c3, ep300, fosb, nr3c1, crp*, and *socs3*) and anti-apoptotic (*bcl2, birc3, grb2*, and *prkcq*) were affected and the net effect to apoptosis is not apparent. The interaction with the AaΔ29Δ29P strain down-regulated transcription of cellular receptors and adhesion, highlighting the *faslg* and *fas* which also participate in pro-apoptotic pathways. Finally, AaΔ29Δ29P upregulated the transcription of *il-1a, il-1*β*, il-6, cxcl-8*, and *tnfrsf1*β, encoding cytokines and chemokines involved in the inflammatory response. The data suggested a potential role of OMP29 and its paralogue to the host response of GECs. Alternatively, the effect may be due to changes in virulence expression of *Aa* after the deletion of *omp29* and *omp29*^*par*^. Further investigation is needed to understand the influence of OMP29 and OMP29^par^ on the host response of GECs to *Aa*. This study did not address the net impact of OMP29 and OMP29^par^ on GECs. We will explore the role and the mechanisms of these proteins in the apoptosis of GECs in future studies.

The study is limited by its *in vitro* assessment of the virulence determinants of the serotype a AaD7S. The innate immune response of epithelial cells may differ when challenged with other *Aa* serotypes with different virulence potentials [37–39]. In addition, we evaluated only the response of epithelial cells monolayers to the bacterial challenges and not the response of the oral mucosa with multiple immune cells. Moreover, given the plethora of microorganisms at the oral epithelium, the overall responses are triggered by the whole community and not by a single species [40].

*Aa* is known to exhibit strain-to-strain variation in virulence. The results of this study did not exclude the possibility that different *Aa* strains may exhibit distinct effects on GECs. However, the effects of OMP29 and OMP29^par^ in different *Aa* strains on GECs are likely similar. First, OMP29 and OMP29^par^ are highly conserved among genetically distanced *Aa* strains (Supplementary Figure 1). Second, the recombinant OMP29 in our study was derived from a serotype b strain HK1651, and exhibited similar inhibitory properties demonstrated in serotype a strain AaD7S that expressed this protein. Finally, a preliminary analysis of the upstream regions of *omp29* and *omp29*^*par*^ did not reveal any apparent structural variations that may lead to differential gene expression (data not shown). Additional studies are needed to resolve this question.

It is worth noting the clinical implication of the inhibition of CXCL-8 by OMP29 of *Aa*. CXCL-8 is a potent chemoattractant for polymorphonuclear cells into the infected tissue [41]. A balanced proportion of neutrophils in the periodontal tissues is essential for homeostasis, and both too high or too low numbers of neutrophils contribute to periodontitis [40]. Thus, the inhibitory activity in GECs response by OMP29 may break this balance, decreasing the numbers of neutrophils at the infected sites, allowing a more permissive niche for bacteria replication. This transient inhibition could delay the recruitment of neutrophils, and therefore facilitate the initial colonization of this pathogen and other microorganisms [42]. Other pathogens such as *Porphyromonas gingivalis* inhibit the production of CXCL-8 in periodontal tissues by secreting a serine phosphatase (SerB) [43]. OMP29 may be an additional tool of *Aa* to alter the microenvironment of subgingival biofilm, allowing bacterial survival and tissue destruction. Also, OMP29 may act in synergy with *Aa* secretin HofQ, which uptakes CXCL-8 [44], limiting CXCL-8 availability in periodontal tissues. These data are in accordance with a study in humans that reported that *Aa*-negative gingival crevicular fluid (GCF) contained higher concentrations of CXCL-8 than sites harboring *Aa* [45].

In conclusion, our study suggested that OMP29 and its paralogue of *Aa* may suppress the expression of CXCL-8, modulate the host responses in epithelial cells, and permit initial colonization of *Aa*.

## Data Availability Statement

The original contributions presented in the study are included in the article/Supplementary Files, further inquiries can be directed to the corresponding author/s.

## Ethics Statement

The animal study was reviewed and approved by Ethics and Research Committee on Animals of the Institute of Biomedical Sciences of the University of São Paulo.

## Author Contributions

CC: supervision, investigation, conceptualization and design of the study, formal analysis, writing original draft, and review and editing. MM: supervision, investigation, conceptualization and design of the study, writing review, and editing. RM: design of the study and writing original draft. GM: formal/statistical analysis, writing original draft, and review and editing. DK and VS: investigation, formal analysis, and writing original draft. EA-S: investigation, formal analysis and writing original draft, and review and editing. SP: investigation and writing original draft. MS: investigation, formal analysis, design of the study, writing original draft, and review and editing. All authors contributed to manuscript revision, read, and approved the submitted version.

## Funding

This study was supported by FAPESP (Grants 2011/18683-1, 2012/05911-9, and 2015/18273-9), CAPES PNPD (Grant 88887.466596/2019-00), and NIH (Grant R01 DE012212).

## Conflict of Interest

The authors declare that the research was conducted in the absence of any commercial or financial relationships that could be construed as a potential conflict of interest.

## Publisher's Note

All claims expressed in this article are solely those of the authors and do not necessarily represent those of their affiliated organizations, or those of the publisher, the editors and the reviewers. Any product that may be evaluated in this article, or claim that may be made by its manufacturer, is not guaranteed or endorsed by the publisher.

## Supplementary Material

Supplementary Figure 1 Multiple Sequence Aligment of OMP29 and OMP29^par^ among *Aa* strains of different serotypes. The multiple sequence alignments of OMP29 **(A)** and OMP29^par^ **(B)** of representative *Aa* strains of serotype a, b and c (HK1651, ANH9381, D7S-1, H5P-1, D11S-1, and D17P-2) was performed with Clustal Omega using the default setting. Each protein demonstrates 99% amino acid sequence identify among strains. These two homologous proteins also share approximately 75% amino acid sequence identify.

Supplementary Figure 2 SDS-PAGE, ELISA and Immunoblot of *Aa* OMP29^His^. **(A)** 10% polyacrylamide gel of SDS-PAGE with aliquot of *Aa* OMP29^His^ (OMP29 recombinant protein of *A. actinomycetemcomitans* HK1651). *E. coli* BL21 (DE3) transformed with pET28B/*omp29* vector was cultured by standard methods for 2 h with 1 mM IPTG for the purpose of protein expression induction. MWM: Molecular Weight Marker - Precision Plus Protein Standard, Bio-Rad, in kDa. The arrow indicates a band corresponding to *Aa* OMP29^His^ heated with approximately 38.44 kDa (predicted by the website www.bioinformatics.org). **(B)** ELISA Optical Density (O.D.) 490nm data at 63 days after primary immunization and booster injections at 7 days, 3, 4, 6 and 7 weeks, using different serum dilutions (1:500, 1:1,000, 1:2,000 and 1:5,000) from immunized mice against *Aa* OMP29^His^ and non-immunized controls. ^*^Two-way ANOVA followed by post-test of Tukey, p < 0.0001. **(C)** 10% polyacrylamide gel of SDS-PAGE with aliquot of *Aa* OMP29^His^. **(D)** *Western Blot:* (D_1_) Detection of OMP29^His^ using mice polyclonal anti-*Aa* OMP29^His^ serum. (D_2_) Detection of *Aa* OMP29^His^ using serum from aggressive periodontitis patient.

Supplementary Figure 3 Growth curve of *A. actinomycetemcomitans* strains. Bacteria grown overnight were diluted in mTSB medium and grown for 16h to obtain the growth curve and the outer membrane extracts. Growth curve: cell density at O.D._420−580_ were determined hourly. Each point represents the mean values of two independent cultures. AaD7S: *A. actinomycetemcomitans* D7S-1 wild-type strain; AaΔ29: *A. actinomycetemcomitans* D7S-1 *omp29* mutant strain; AaΔ29P: *A. actinomycetemcomitans* D7S-1 *omp29*^*par*^ mutant strain; AaΔ29Δ29P: *A. actinomycetemcomitans* D7S-1 *omp29* and *omp29*^*par*^ mutant strain; mTSB: media not inoculated was used as a control. The growth rates for each strain in the exponential phase were 1.2 h^−1^, 1.04 h^−1^, 1.03 h^−1^, 1.02 h^−1^ and 0.92 h^−1^ for AaD7S, AaΔ29, AaΔ29P and AaΔ29Δ29P, respectively.

Supplementary Table 1 Identification and the amounts of number of peptide spectrum matches (PSM) of proteins with high scoring peptides from gel slices number 1–12, according to Figure 1A.

Supplementary Table 2 Function of the statistically relevant genes analyzed by the microarray assay.
