# Supplementary figures and images for: Aggregatibacter actinomycetemcomitans Outer Membrane Proteins 29 and 29 Paralogue Induce Evasion of Immune Response
Source: Front Oral Health. 2022 Feb 3;3:835902. doi: 10.3389/froh.2022.835902 (PMC8851312; doi:10.3389/froh.2022.835902)

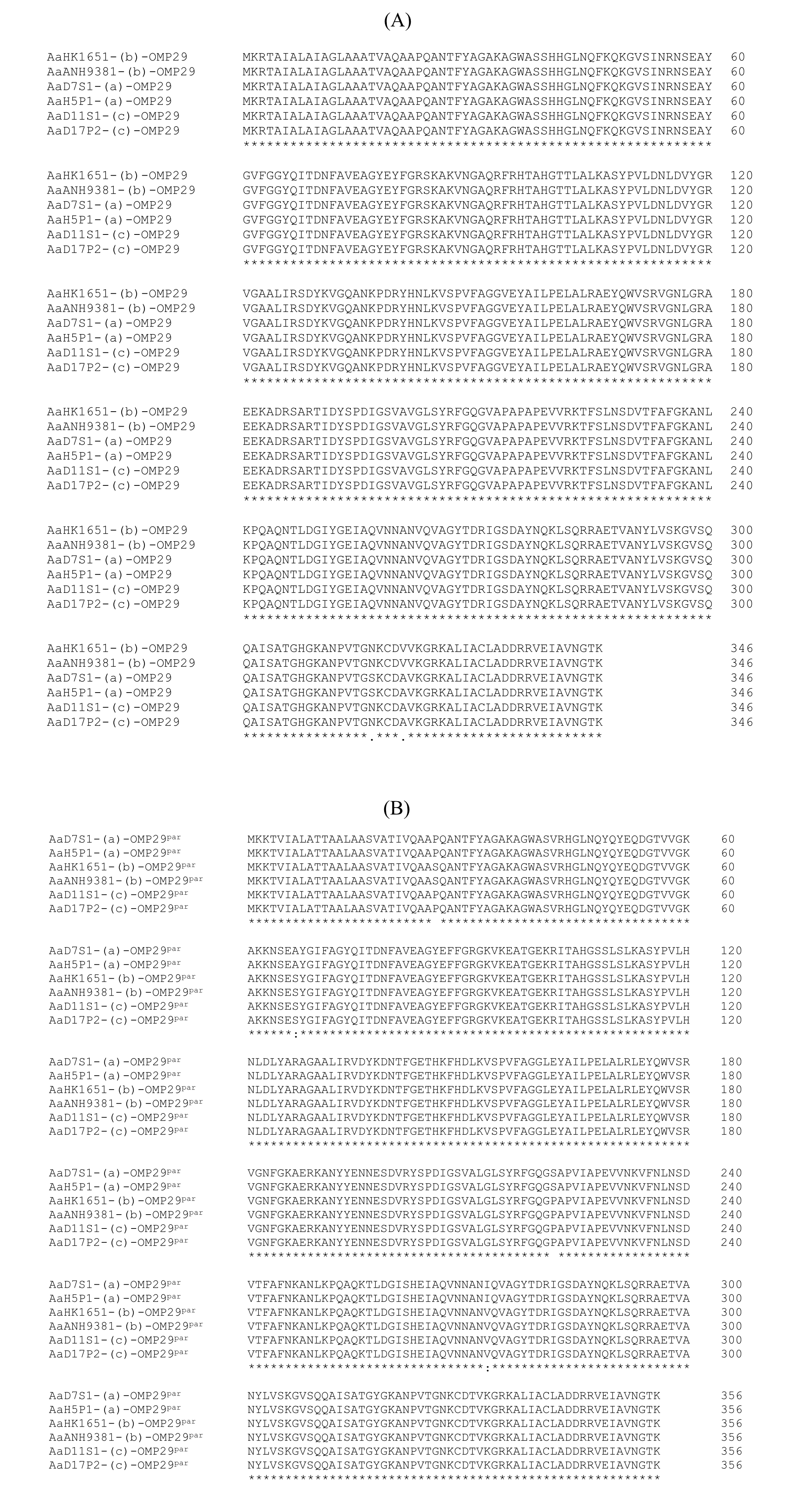

Supplement: Supplementary Figure 1 — Multiple Sequence Aligment of OMP29 and OMP29par among Aa strains of different serotypes. The multiple sequence alignments of OMP29 (A) and OMP29par (B) of representative Aa strains of serotype a, b and c (HK1651, ANH9381, D7S-1, H5P-1, D11S-1, and D17P-2) was performed with Clustal Omega using the default setting. Each protein demonstrates 99% amino acid sequence identify among strains. These two homologous proteins also share approximately 75% amino acid sequence identify. [file Image_1.TIFF]

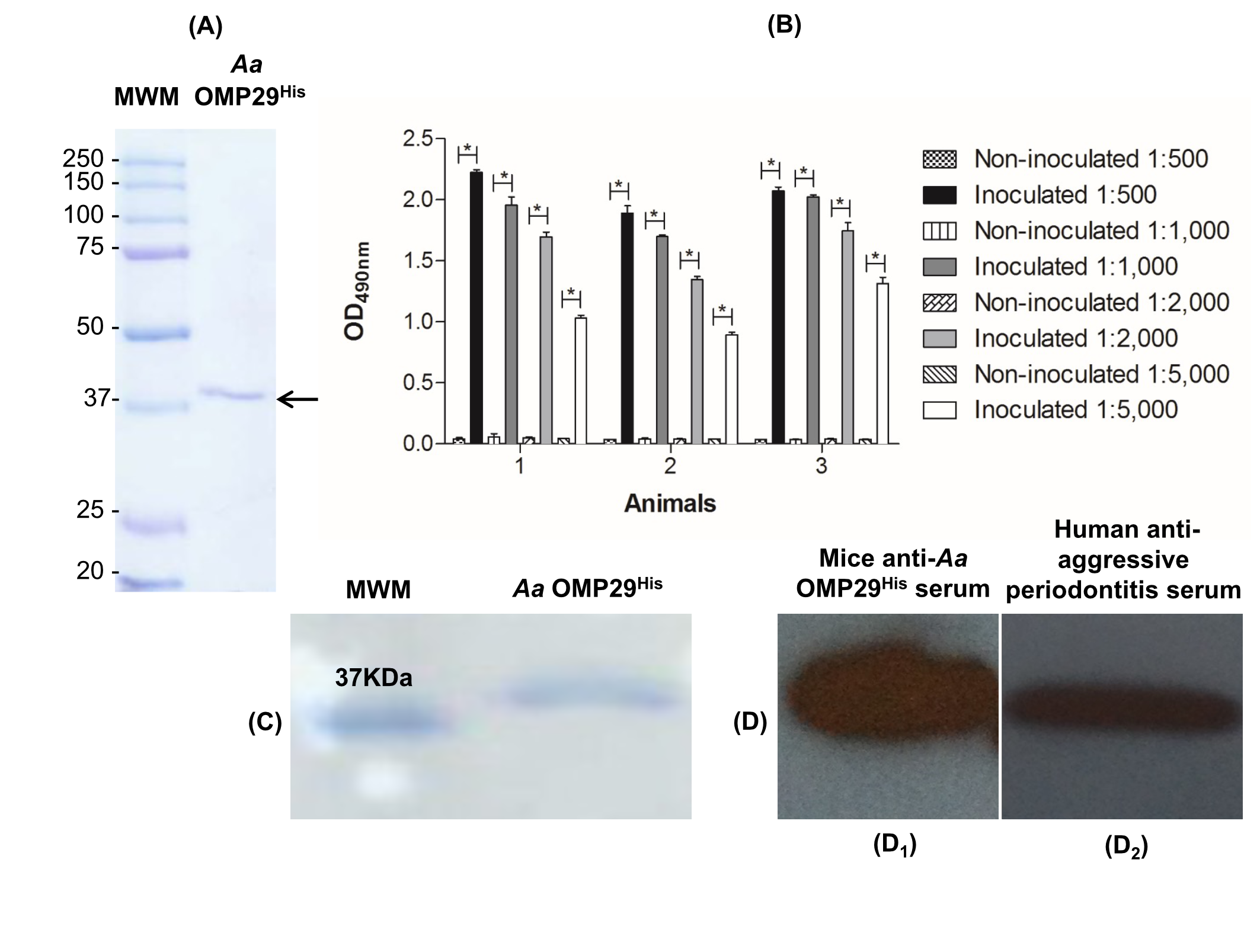

Supplement: Supplementary Figure 2 — SDS-PAGE, ELISA and Immunoblot of Aa OMP29His. (A) 10% polyacrylamide gel of SDS-PAGE with aliquot of Aa OMP29His (OMP29 recombinant protein of A. actinomycetemcomitans HK1651). E. coli BL21 (DE3) transformed with pET28B/omp29 vector was cultured by standard methods for 2 h with 1 mM IPTG for the purpose of protein expression induction. MWM: Molecular Weight Marker - Precision Plus Protein Standard, Bio-Rad, in kDa. The arrow indicates a band corresponding to Aa OMP29His heated with approximately 38.44 kDa (predicted by the website www.bioinformatics.org). (B) ELISA Optical Density (O.D.) 490nm data at 63 days after primary immunization and booster injections at 7 days, 3, 4, 6 and 7 weeks, using different serum dilutions (1:500, 1:1,000, 1:2,000 and 1:5,000) from immunized mice against Aa OMP29His and non-immunized controls. *Two-way ANOVA followed by post-test of Tukey, p < 0.0001. (C) 10% polyacrylamide gel of SDS-PAGE with aliquot of Aa OMP29His. (D) Western Blot: (D1) Detection of OMP29His using mice polyclonal anti-Aa OMP29His serum. (D2) Detection of Aa OMP29His using serum from aggressive periodontitis patient. [file Image_2.TIFF]

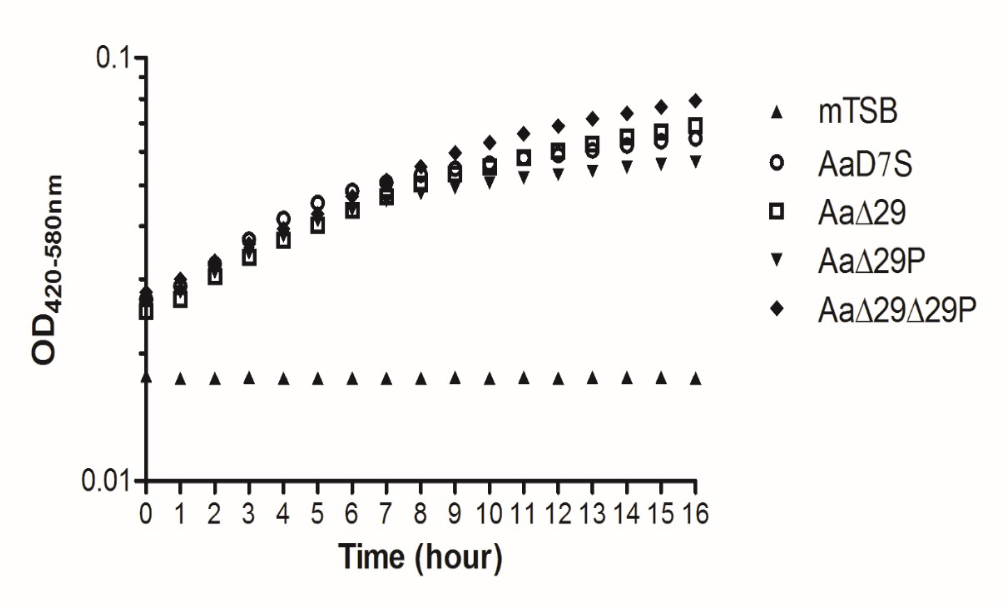

Supplement: Supplementary Figure 3 — Growth curve of A. actinomycetemcomitans strains. Bacteria grown overnight were diluted in mTSB medium and grown for 16h to obtain the growth curve and the outer membrane extracts. Growth curve: cell density at O.D.420−580 were determined hourly. Each point represents the mean values of two independent cultures. AaD7S: A. actinomycetemcomitans D7S-1 wild-type strain; AaΔ29: A. actinomycetemcomitans D7S-1 omp29 mutant strain; AaΔ29P: A. actinomycetemcomitans D7S-1 omp29par mutant strain; AaΔ29Δ29P: A. actinomycetemcomitans D7S-1 omp29 and omp29par mutant strain; mTSB: media not inoculated was used as a control. The growth rates for each strain in the exponential phase were 1.2 h−1, 1.04 h−1, 1.03 h−1, 1.02 h−1 and 0.92 h−1 for AaD7S, AaΔ29, AaΔ29P and AaΔ29Δ29P, respectively. [file Image_3.TIFF]
