# Supplementary material for: Aggregatibacter actinomycetemcomitans Outer Membrane Proteins 29 and 29 Paralogue Induce Evasion of Immune Response
Source: Front Oral Health. 2022 Feb 3;3:835902. doi: 10.3389/froh.2022.835902 (PMC8851312; doi:10.3389/froh.2022.835902)
Supplement: Supplementary Table 1 — Identification and the amounts of number of peptide spectrum matches (PSM) of proteins with high scoring peptides from gel slices number 1–12, according to Figure 1A. [file Table_1.pdf]

Supplementary Material

Supplementary Tables

Supplementary Table 1. Identification and the amounts of number of peptide spectrum matches (PSM) of proteins with high scoring peptides from gel slices number 1-12, according to Figure 1A.

Samples	Acession number	Protein Description	#PSM
**Slice #1 AaD7S cfngcx.**	387121739	outer membrane protein 39	40
	387120192	outer membrane protein 29	20
	387121153	flp operon protein D	19
	387120329	CRISPR-associated protein	21
	387121156	flp operon protein C	4
	387121155	type II/IV secretion system secretin RcpA/CpaC	3
	387121481	ornithine carbamoyltransferase	1
**Slice #2 AaD7S**	387120192	outer membrane protein 29	187
	387120232	outer membrane protein 29 paralogue	21
	387121156	flp operon protein C	12
	387121456	glyceraldehyde-3-phosphate dehydrogenase, type I	9
	387120698	translation elongation factor Tu	7
	387120045	malate dehydrogenase	8
	387121739	outer membrane protein 39	5
	387121642	6-phosphofructokinase	2
	387121173	transaldolase B	2
**Slice #3 AaD7S**	387121156	flp operon protein C	37
	387120192	outer membrane protein 29	26
	387120232	outer membrane protein 29 paralogue	17
	387121739	outer membrane protein 39	503

**Slice #4 AaΔ29Δ29P**	387121153	flp operon protein D	23
	387121804	spermidine/putrescine-binding periplasmic protein 1	15
	387120698	translation elongation factor Tu	14
	387120329	CRISPR-associated protein	14
	387121016	putative GDP-mannose dehydratase	7
	387122049	maltoporin	6
	387120252	peptide methionine sulfoxide reductase MsrA/msrB	8
	387120595	glycerophosphoryl diester phosphodiesterase	7
	387121567	L-asparaginase	4
	387121138	protein RecA	4
	387122047	maltose ABC transporter periplasmic protein	3
	387120423	LOW QUALITY PROTEIN: outer membrane protein 64, partial	3
	387121146	Flp pilus assembly protein TadG	3
	387120319	oxidoreductase domain-containing protein	3
	387120619	fructose-bisphosphate aldolase	2
	387120387	serine hydroxymethyltransferase	3
	387120154	transhydrogenase	2
	387120671	outer membrane protein assembly complex, YaeT protein	2
	387120283	L-lactate dehydrogenase LctD	2
	387119893	UspA protein	4
	387121712	membrane-bound lytic murein transglycosyllase A	3
	387120590	rod shape-determining protein MreB	1
	387121722	extracellular solute-binding protein	2
**Slice #5 AaΔ29Δ29P**	387121739	outer membrane protein 39	111
	387121679	NlpB protein	22
	387120698	translation elongation factor Tu	17
	387122041	Fe3+ ABC transporter, iron-binding protein	11
	387120972	transporter	12

	387121712	membrane-bound lytic murein transglycosyllase A	10
	387121792	thiamin/thiamine pyrophosphate ABC transporter	8
	387120268	UDP-glucose 4-epimerase	5
	387120671	outer membrane protein assembly complex, YaeT protein	7
	387121456	glyceraldehyde-3-phosphate dehydrogenase, type I	8
	387121723	cystathionine beta-lyase	11
	387121156	flp operon protein C	4
	387121142	transaldolase	4
	387121091	glycyl-tRNA synthetase subunit alpha	3
	387122004	putative TdeA	5
	387121567	L-asparaginase	4
	387120252	peptide methionine sulfoxide reductase MsrA/msrB	3
	387120242	S49 family peptidase	2
	387121173	transaldolase B	2
	387121642	6-phosphofructokinase	3
	387120783	membrane-bound lytic murein transglycosyllase C	2
	387121153	flp operon protein D	2
	387121710	high-affinity zinc uptake system protein ZnuA	4
	387121155	type II/IV secretion system secretin RcpA/CpaC	1
	387121532	4-hydroxy-3-methylbut-2-enyl diphosphate reductase	1
	387121714	fructose-1,6-bisphosphatase	1
**Slice #6 AaΔ29Δ29P**	387121739	outer membrane protein 39	48
	387121156	flp operon protein C	20
	387120698	translation elongation factor Tu	13
	387120799	elongation factor EF1B	11
	387120262	hypothetical protein D7S_00474	9
	387121642	6-phosphofructokinase	6
	387121456	glyceraldehyde-3-phosphate dehydrogenase, type I	7

	387121679	NlpB protein	4
	387119892	universal stress protein UspE	2
	387120671	outer membrane protein assembly complex, YaeT protein	2
	387120456	cysteinyl-tRNA synthetase	2
	387121153	flp operon protein D	1
	387120252	peptide methionine sulfoxide reductase MsrA/msrB	1
	387122050	maltose operon periplasmic protein	2
**Slice #7 AaΔ29**	387121739	outer membrane protein 39	100
	387121153	flp operon protein D	15
	387120232	outer membrane protein 29 paralogue	10
	387120329	CRISPR-associated protein	10
	387120698	translation elongation factor Tu	6
	387121016	putative GDP-mannose dehydratase	2
	387121146	Flp pilus assembly protein TadG	5
	387122047	maltose ABC transporter periplasmic protein	4
	387120319	oxidoreductase domain-containing protein	3
	387120252	peptide methionine sulfoxide reductase MsrA/msrB	4
	387121804	spermidine/putrescine-binding periplasmic protein 1	2
	387120283	L-lactate dehydrogenase LctD	3
	387121156	flp operon protein C	2
	387120328	CRISPR-associated Csy2 family protein	2
	387121138	protein RecA	2
	387120595	glycerophosphoryl diester phosphodiesterase	2
	387119877	phenylalanyl-tRNA synthetase subunit alpha	1
**Slice #8 AaΔ29**	387120232	outer membrane protein 29 paralogue	19
	387121739	outer membrane protein 39	21
	387120698	translation elongation factor Tu	13
	387121679	NlpB protein	5

	387120972	transporter	4
	387121712	membrane-bound lytic murein transglycosyllase A	4
	387122041	Fe3+ ABC transporter, iron-binding protein	3
	387120384	hemolysin A	2
	387121710	high-affinity zinc uptake system protein ZnuA	2
	387121567	L-asparaginase	4
	387120242	S49 family peptidase	4
	387120252	peptide methionine sulfoxide reductase MsrA/msrB	3
	387121456	glyceraldehyde-3-phosphate dehydrogenase, type I	2
	387121156	flp operon protein C	2
	387121801	C4-dicarboxylate membrane transporter	3
	387120329	CRISPR-associated protein	2
	387121792	thiamin/thiamine pyrophosphate ABC transporter	3
**Slice #9 AaΔ29**	387120232	outer membrane protein 29 paralogue	145
	387121156	flp operon protein C	19
	387120698	translation elongation factor Tu	16
	387121456	glyceraldehyde-3-phosphate dehydrogenase, type I	20
	387120045	malate dehydrogenase	6
	387121739	outer membrane protein 39	14
	387121642	6-phosphofructokinase	6
	387122063	HflC protein	6
	387120384	hemolysin A	4
	387121173	transaldolase B	3
	387119892	universal stress protein UspE	8
	387121874	thioredoxin-disulfide reductase	3
	387121801	C4-dicarboxylate membrane transporter	2
	387120262	hypothetical protein D7S_00474	2
	387121679	NlpB protein	2

	387120569	magnesium and cobalt transport protein CorA	22
	387120281	ribose-Phosphate pyrophosphokinase	2
	387120799	elongation factor EF1B	4
	387121775	iron ABC superfamily ATP binding cassette transporter, binding protein	2
	387121635	signal peptidase I	2
	387121648	formate acetyltransferase	1
	387120456	cysteinyl-tRNA synthetase	1
**Slice #10 AaΔ29P**	387121739	outer membrane protein 39	203
	387120192	outer membrane protein 29	25
	387121155	type II/IV secretion system secretin RcpA/CpaC	20
	387121153	flp operon protein D	9
	387120329	CRISPR-associated protein	13
	387120154	transhydrogenase	7
	387120595	glycerophosphoryl diester phosphodiesterase	6
	387120232	outer membrane protein 29 paralogue	6
	387122049	maltoporin	7
	387121804	spermidine/putrescine-binding periplasmic protein 1	6
	387120283	L-lactate dehydrogenase LctD	9
	387121156	flp operon protein C	5
	387120328	CRISPR-associated Csy2 family protein	5
**Slice #11 AaΔ29P**	387120192	outer membrane protein 29	351
	387121679	NlpB protein	30
	387121156	flp operon protein C	28
	387121456	glyceraldehyde-3-phosphate dehydrogenase, type I	17
	387121739	outer membrane protein 39	14
	387120698	translation elongation factor Tu	7
	387121149	Flp pilus assembly protein TadD	3
	387121155	type II/IV secretion system secretin RcpA/CpaC	8

	387120242	S49 family peptidase	5
	387121173	transaldolase B	4
	387121642	6-phosphofructokinase	3
	387119892	universal stress protein UspE	2
	387121712	membrane-bound lytic murein transglycosyllase A	2
	387120972	transporter	1
	387121001	chaperonin GroL	1
**Slice #12 AaΔ29P**	387120192	outer membrane protein 29	115
	387121156	flp operon protein C	53
	387121739	outer membrane protein 39	27
	387121155	type II/IV secretion system secretin RcpA/CpaC	12
	387121149	Flp pilus assembly protein TadD	5
	387121456	glyceraldehyde-3-phosphate dehydrogenase, type I	7
	387121357	lipoprotein	5
	387121874	thioredoxin-disulfide reductase	4
	387120799	elongation factor EF1B	6
	387121173	transaldolase B	4
	387122063	HflC protein	3
	387121775	iron ABC superfamily ATP binding cassette transporter, binding protein	5
	387120698	translation elongation factor Tu	3
	387121001	chaperonin GroL	2
