# Supplementary material for: Aggregatibacter actinomycetemcomitans Outer Membrane Proteins 29 and 29 Paralogue Induce Evasion of Immune Response
Source: Front Oral Health. 2022 Feb 3;3:835902. doi: 10.3389/froh.2022.835902 (PMC8851312; doi:10.3389/froh.2022.835902)
Supplement: Supplementary Table 2 — Function of the statistically relevant genes analyzed by the microarray assay. [file Table_2.pdf]

frontiers

Supplementary Table 2. Function of the statistically relevant genes analyzed by the microarray assay

Gene	Gene name	Function of genes involved in apoptosis and anti-apoptotic mechanisms
bcl2	B-cell lymphoma 2	Generally, the BCL-2 family of proteins, promote the irreversible release of intermembrane space proteins by regulating mitochondrial outer membrane permeabilization. Thereafter, this death control mechanism, activate caspase and apoptosis pathways.
birc3	Baculoviral IAP repeat-containing protein 3	One of the pro-survival factors involved in transcriptional activation of the NF-kB pathway, the third major anti-apoptotic pathway.
casp3	Caspase 3	Involvement in the proteolytic cleavage of the apoptotic process, beyond the cleavage of proteins involved in DNA repair, cell cycle, cytoskeletal and nuclear organization.
c3	Complement componente 3	Central pivot of the complement system involved in membrane attack and responsible for lytic activity in pathogens.
crp	C-reactive protein	A classic acute-phase protein, bound to apoptotic cells in a Ca2+-dependent manner and augmented the classical pathway of complement activation.
ep300	E1A binding protein p300	It is a transcription cofactor with intrinsic acetyltransferase activity. It plays a crucial role in proliferation and apoptosis during embryogenesis. Catalyzes histone acetylation and transcription factors, and it plays a role in epigenetic regulation. It is abnormal function may result in inflammation.
fosb	FBJ murine osteosarcoma viral oncogene homolog B	It induces the activation of human T-cell death, when occurs dimerization of c-Jun mediated via T cell receptor/Cluster of differentiation.
grb2	Growth factor receptor-bound protein 2	It contributes to proliferation, normal cell development, binding other proteins to the membrane, after the recruitment of epidermal growth factor receptor (EGFR) activated or receptor tyrosine kinase (RTK).
nr3c1	Nuclear receptor subfamily 3, group C, member 1	It has numerous suppressing effects on immune cells. It inhibits the function of both activating protein-1 (AP1) family members and NF-kB, and thus suppress a large number of responses to pro-inflammatory signaling pathways.
prkcq	Protein kinase C-theta	It activates T cells and T cell receptor stimulation, cell proliferation, IL-2 production, and anti-apoptotic mechanisms.

socs3	Suppressors of cytokine signaling protein 3	It plays a critical role in modulating signaling cytokines promoting inflammation and apoptosis via JAK/STAT.
**Gene**	**Gene name**	**Function of the celular receptors and adhesin genes**
cd74	Cluster of differentiation 74	Chaperone molecules of the major histocompatibility complex type II and costimulatory molecule immune cells. The expression of CD74 can occur regardless of MHC II, and additionally acts as cell surface receptor for macrophage migration inhibitory factor (MIF), receptor for *Helicobacter pylori* facilitating adherence of the pathogens to epithelial cells gastric and, accessory signaling molecules in cell proliferation processes.
fas	FAS receptor	Receptor associated with activation-induced cell death.
faslg	FAS ligand	FAS cognate receptor associated with activation-induced cell death.
trl1	Toll-like receptor 1	Recognizes peptidoglycan and lipoproteins with Toll-like receptor 2.
tnfrs1β	Tumor Necrosis Factor Receptor Superfamily, Member 1B	Transmembrane glycoprotein that induces apoptosis or cell survival. The binding of TNF to its cognate receptors initiates the assembly of signaling complexes associated with the receptor, stimulates the activation of multiple signaling pathways, including: the nuclear factor kB (NF-kB), kinase c-Jun N-terminal (C -Jun N-terminal kinase, JNK) and mitogen-activated protein kinase p38 (MAPKs), and in some cases, cell death.
vcam1	Vascular cell adhesion protein 1	A adhesive cell surface glycoproteins for certain leukocytes and tumor cells and induces endothelial cell surface molecules mediating intercellular adhesion through interaction with the integrin very late antigen-4 (VLA4) which is expressed in monocytes, lymphocytes, basophils, eosinophils and certain tumor cells, but not neutrophils.
**Gene**	**Gene name**	**Function of the pro-inflammatory cytokines genes**
il-1α	Interleukin 1α	A critical early mediator of inflammatory responses triggering vasodilatation and attracting monocytes and neutrophils (leukocytes) to sites of tissue damage and stress.
il-1β	Interleukin 1β	An important pro-inflammatory cytokine involved in the increasing the expression of molecules that mediate adhesion of leukocytes to the site of inflammation.

il-6	Interleukin 6	IL-6 with IL-6R and the receptor gp 130, activate the intracellular phosphorylation of a protein called Janus kinase (Jak), which activates the activator of transcription (STAT-3). STAT-3 enters into the nucleus and activates the expression of target genes.
cxcl-8	C-X-C- Motif Chemokine Ligand 8	Neutrophil-attracting chemokine that activates high affinity chemokine receptors 1and 2.
